# A Multiwell-Based Assay for Screening Thyroid Hormone Signaling Disruptors Using *thibz* Expression as a Sensitive Endpoint in *Xenopus laevis*

**DOI:** 10.3390/molecules27030798

**Published:** 2022-01-25

**Authors:** Jinbo Li, Yuanyuan Li, Min Zhu, Shilin Song, Zhanfen Qin

**Affiliations:** 1 State Key Laboratory of Environmental Chemistry and Ecotoxicology, Research Center for Eco-Environmental Sciences, Chinese Academy of Sciences, Beijing 100085, China; jbli2016_st@rcees.ac.cn (J.L.); yyli@rcees.ac.cn (Y.L.); zhumin2@jshb.gov.cn (M.Z.); songshilin21@mails.ucas.ac.cn (S.S.); 2 University of Chinese Academy of Sciences, Beijing 100049, China

**Keywords:** thyroid hormone, screening assay, *Xenopus laevis*, *thibz* gene, multiwell plate

## Abstract

There is a need for rapidly screening thyroid hormone (TH) signaling disruptors in vivo considering the essential role of TH signaling in vertebrates. We aimed to establish a rapid in vivo screening assay using *Xenopus laevis* based on the T3-induced *Xenopus* metamorphosis assay we established previously, as well as the *Xenopus* Eleutheroembryonic Thyroid Assay (XETA). Stage 48 tadpoles were treated with a series of concentrations of T3 in 6-well plates for 24 h and the expression of six TH-response genes was analyzed for choosing a proper T3 concentration. Next, bisphenol A (BPA) and tetrabromobisphenol A (TBBPA), two known TH signaling disruptors, were tested for determining the most sensitive TH-response gene, followed by the detection of several suspected TH signaling disruptors. We determined 1 nM as the induction concentration of T3 and *thibz* expression as the sensitive endpoint for detecting TH signaling disruptors given its highest response to T3, BPA, and TBBPA. And we identified betamipron as a TH signaling agonist, and 2,2′,4,4′-tetrabromodiphenyl ether (BDE-47) as a TH signaling antagonist. Overall, we developed a multiwell-based assay for rapidly screening TH signaling disruptors using *thibz* expression as a sensitive endpoint in *X. laevis*.

## 1. Introduction

Thyroid hormones (THs, T3 and T4) produced by the thyroid gland are critical for growth and development in vertebrates [1]. THs exert their genomic actions principally through TH signaling, in which THs bind to their receptors (TR), resulting in the recruitment of coregulatory proteins to form complexes, thereby regulating target gene expression by binding to DNA sequences in the promoter of target genes [2,3]. Unfortunately, some endocrine-disrupting chemicals (EDCs) could interfere with TH signaling as well as TH production, metabolism, and transportation. TH signaling disruptors have been demonstrated to disrupt TH signaling through interacting with TR or other components in TH signaling [4], possibly leading to adverse effects on the growth and development in vertebrates [5,6,7]. For instance, bisphenol A (BPA) and tetrabromobisphenol A (TBBPA) are known TH signaling antagonists that disrupt the binding of THs to TR and affect TH-dependent development in *Xenopus* [8,9,10,11], but they also exhibit TH signaling agonism in certain situations [12,13].

Several in vitro binding assays [14] and in vitro transfection assays based on reporter genes [15] have been developed to detect TH signaling disruptors. In vitro TR binding assays can detect whether chemicals bind to TR, but cannot distinguish whether they are agonists or antagonists of TH signaling. In contrast, in vitro transfection assays can screen TH signaling agonists or antagonists but not completely reflect what actually happens in vivo due to the lack of the capacity to metabolize test chemicals in cells. Nevertheless, the reporter-enzymes-based GH3-TRE-Luciferase assay established by Freitas et al. (2011) [12] has been modified into a high throughput screening assay in the Tox21 project, aiming to screen TH signaling agonists or antagonists. However, it is still necessary to develop rapid in vivo screening assays for detecting TH signaling disruptors.

TH-dependent amphibian metamorphosis offers an opportunity to investigate thyroid disruption including TH signaling disruption in vivo [16,17]. Specifically, the amphibian metamorphosis assay (AMA) [18] has been designed to identify substances with thyroid activity and the interpretation of the AMA may be complicated due to multiple mechanisms of thyroid disruption and the possible involvement of other pathways besides the thyroid system. Furthermore, Fini et al. (2007) [19] established the *Xenopus* Eleutheroembryonic Thyroid Assay (XETA) [20], which has been validated and issued by the Organization for Economic Co-operation and Development (OECD). In the XETA, stage 45 fluorescent transgenic *X. laevis* bearing a TH/bZIP-GFP construct are exposed to chemicals in 6-well plates for 72 h with or without 5 nM T3, and fluorescent intensity is detected to assess the thyroid disrupting activity of the test chemicals. However, the TH/bZIP-GFP transgenic line is unavailable for many laboratories, which restricts the application of the XETA. Previously, we developed a T3-induced *Xenopus* metamorphosis assay (TIXMA) for the detection of TH signaling disruptors [21,22]. In the TIXMA, stage 52 tadpoles are exposed to chemicals in the absence or presence of 1 nM T3 in glass tanks containing 4 L, and TH-response gene expression after 24-h exposure and gross morphology after 96-h exposure are measured. Relative to the XETA employing 6-well plates, the TIXMA requires a large amount of water, especially due to daily renewal, resulting in heavy labor and discharge of lots of chemical-contaminated experimental water. To sum up, a simpler and more rapid universally available in vivo screening assay is still needed for detecting TH signaling disruptors.

The present study aimed to develop a simple and rapid screening assay for detecting TH signaling disruptors using wild-type *Xenopus* tadpoles, referencing the XETA and the TIXMA. Considering their high sensitivity to exogenous TH due to the high expression of the *thyroid hormone receptor (thr)* gene [23,24] and little capability to respond to chemicals that directly or indirectly disrupt TH synthesis due to undeveloped thyroid glands [25,26], tadpoles at stage 48 were employed as test organisms. Tadpoles were exposed to T3 or chemicals or their combination in 6-well plates for 24 h, thereby facilitating rapid and easy screening of TH signaling disruption and reducing possible interferences resulting from unknown mechanisms. Based on the effects of a series of concentrations of T3 on TH-response gene expression, we determined an appropriate concentration of T3 to induce expression of TH-response genes, among which the most sensitive TH-response gene was chosen as the endpoint. Subsequently, we assessed the TH signaling disrupting activity of several suspected TH signaling disruptors, including betamipron, 2,2′,4,4′-tetrabromodiphenyl ether (BDE-47), triclocarban (TCC), triclosan (TCS), benzophenone (BP), and benzophenone-3 (BP-3). Betamipron, a drug to reduce nephrotoxicity [27], was reported as a TR agonist in the Tox21 project [28] and requires testing in vivo to confirm the TR agonist. TCC [29] and TCS [30] are two bacteriostatic agents in personal care products, and BP [31] and BP-3 [32] are two UV filters widely used, with BDE-47 [33] as a brominated retardant. These chemicals were reported to have the potential to interfere with the thyroid system, but whether they affect TH signaling is not yet well-documented. Overall, our study is expected to provide a simple and rapid in vivo assay for screening TH signaling disruptors.

## 2. Results

### 2.1. Determination of T3 Induction Concentration

**Figure 1:**
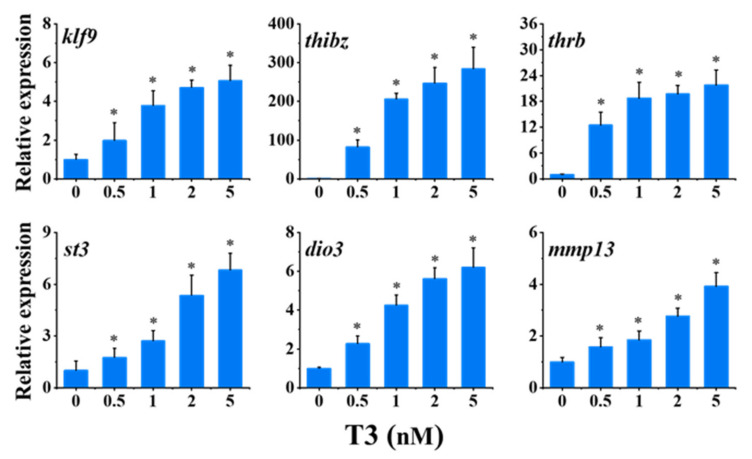
Relative expression of thyroid hormone (TH)-response genes in stage 48 *Xenopus* tadpoles following 24-h exposure to T3. Data are shown as mean ± SEM (*n* = 3). * indicates significant differences between T3 exposure and the control (*p* < 0.05). *klf9: Kruppel-like factor 9; thibz: thyroid hormone induced bZip protein; thrb: thyroid hormone receptor beta; st3: stromelysin-3; dio3: type 3 deiodinase; mmp13: matrix metallopeptidase 13*.

The effects of a series of concentrations of T3 (0.5, 1, 2, and 5 nM) on the expression of six TH-response genes were investigated. As shown in Figure 1, all concentrations of T3, including the lowest concentration, significantly up-regulated the expression of six TH-response genes, *kruppel-like factor 9 (klf9)*, *thyroid hormone induced bZip protein (thibz)*, *thyroid hormone receptor beta (thrb), stromelysin-3 (st3)*, *type 3 deiodinase (dio3)*, and *matrix metallopeptidase 13 (mmp13)*, in a concentration-dependent manner. The changes of *klf9*, *thibz*, *thrb*, *st3*, *dio3*, and *mmp13* in the highest group of T3 (5 nM) were about 5-, 280-, 21-, 6-, 6-, and 4-fold of the control, respectively. Moreover, among the six TH-response genes, *thibz* was the most sensitive, with a 200-fold change following 1 nM T3 treatment. Therefore, 1 nM was selected as the induction concentration of T3 in the following tests.

### 2.2. Antagonistic Actions of BPA and TBBPA on T3-Induced Gene Expression

**Figure 2:**
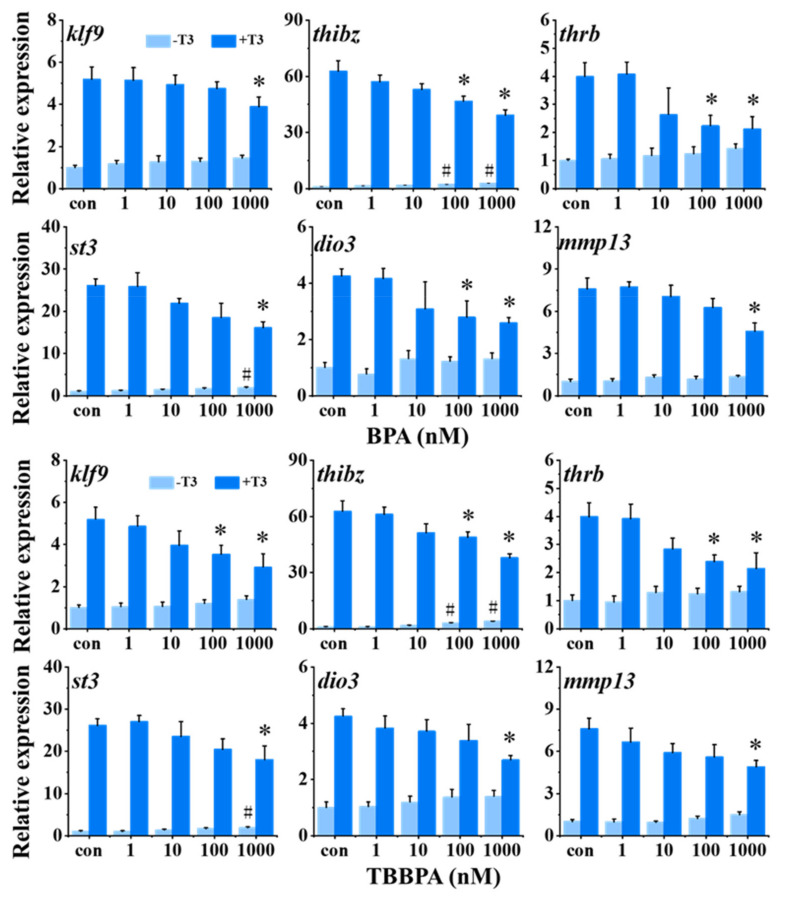
Relative expression of thyroid hormone (TH)-response genes in stage 48 *Xenopus* tadpoles following 24-h exposure to bisphenol A (BPA) and tetrabromobisphenol A (TBBPA) in the absence or presence of 1 nM T3. Data are shown as mean ± SEM (*n* = 3). * and # indicate significant differences between BPA or TBBPA exposure and the control and between BPA or TBBPA + T3 and T3 treatment, respectively (*p* < 0.05). *klf9: Kruppel-like factor 9; thibz: thyroid hormone induced bZip protein; thrb: thyroid hormone receptor beta; st3: stromelysin-3; dio3: type 3 deiodinase; mmp13: matrix metallopeptidase 13*.

The expression of six TH-response genes was measured in tadpoles with BPA or TBBPA exposure in the absence or presence of 1 nM T3. As shown in Figure 2, in the absence of T3, 100 and 1000 nM BPA and TBBPA exposure significantly promoted *thibz* expression. Then, 1000 nM BPA and TBBPA significantly upregulated the expression of *st3*. However, there were no obvious effects on other genes, *klf9*,* thrb*,* dio3*, and *mmp13*. *thibz* expression was significantly induced by as low as 100 nM BPA and TBBPA, indicating that *thibz* was the most sensitive gene among six TH-response genes. Furthermore, T3-induced gene expression of *thibz*, *thrb*, and *dio3* were significantly inhibited by 100 nM BPA, and 1000 nM BPA significantly downregulated all six T3-induced TH-response genes expression. Similar to BPA, 100 nM TBBPA antagonized the T3-induced gene expression of *klf9*, *thibz*, and *thrb*, and 1000 nM TBBPA significantly antagonized all T3-induced genes we tested. In the co-exposure groups, only *thibz* and *thrb* were significantly antagonized by both 100 nM BPA and TBBPA, and T3-induced expression of the other four TH-response genes was antagonized by the higher concentrations of BPA and TBBPA. Taken together, *thibz* expression was chosen as a sensitive endpoint for screening TH signaling disruptors.

### 2.3. TH Signaling Disrupting Activity of Several Suspected TH Signaling Disruptors

**Figure 3:**
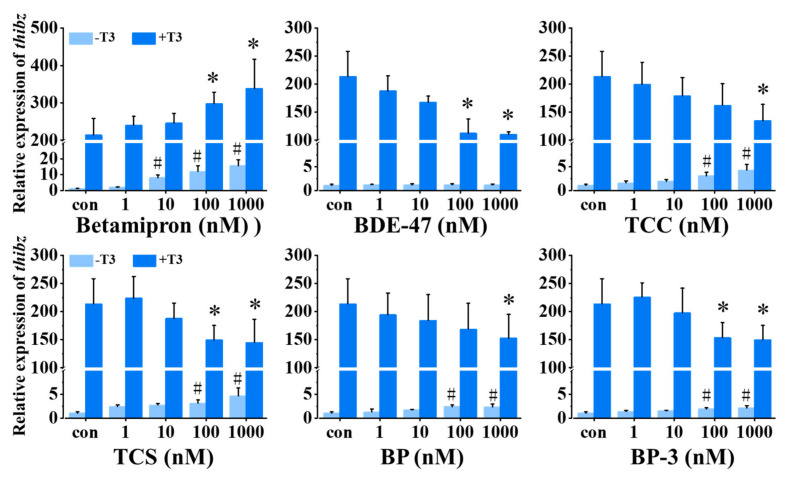
Relative expression of *thibz (thyroid hormone induced bZip protein)* in stage 48 *Xenopus* tadpoles following 24-h exposure to betamipron, 2,2′,4,4′-tetrabromodiphenyl ether (BDE-47), triclocarban (TCC), triclosan (TCS), benzophenone (BP), and benzophenone-3 (BP-3) in the absence or presence of 1 nM T3. Data are shown as mean ± SEM (*n* = 3). * and # indicate significant differences between the chemical exposure and the control and between chemical + T3 and T3 treatment, respectively (*p* < 0.05).

We analyzed the TH signaling disruption activity of betamipron, BDE-47, TCC, TCS, BP, and BP-3 using *thibz* expression as an endpoint. As shown in Figure 3, 1 nM T3 significantly increased the expression of *thibz* expression compared with the control. In the absence of T3, 10–1000 nM betamipron significantly promoted the expression of *thibz* in a concentration-dependent manner, and in the presence of T3, 100–1000 nM betamipron increased the expression of *thibz* compared with the T3 group. BDE-47 inhibited the T3-induced *thibz* expression but did not affect *thibz* expression in the absence of T3. TCC, TCS, BP, and BP-3 had a similar mode of action on *thibz* expression both in the absence and presence of T3. These four chemicals at high concentrations significantly resulted in down-regulation of T3-induced expression of *thibz* expression and high concentrations of these four chemicals alone increased *thibz* expression.

## 3. Discussion

We developed a multiwell-based screening assay to detect TH signaling disruptors based on *thibz* expression analysis using *Xenopus* tadpoles at stage 48. The concentration-dependent upregulation of all tested TH-response gene expressions by T3 treatment has demonstrated the sensitivity of stage 48 tadpoles to T3 within 24 h. Given that 1 nM T3 dramatically induced upregulation of TH-response gene expression but did not reach the expression climax, we determined 1 nM as the induction concentration of T3, avoiding the possibility that the higher concentrations of T3 would cover up the antagonistic actions of tested chemicals. When 1 nM T3 upregulated the expression of all the test TH-response genes, 1000 and/or 100 nM both BPA and TBBPA antagonized T3 actions, showing that this screening assay is effective in detecting TH signaling antagonists. As mentioned above, the XETA employs transgenic TH/bZIP-GFP tadpoles to indicate TH signaling disruption by measuring GFP fluorescence. Considering the finding that *thibz* expression was the most sensitive to T3 and highly responsive to BPA and TBBPA, we chose *thibz* expression as a sensitive endpoint to detect TH signaling disruptors. To our current understanding, *thibz* is believed to be a gene that specifically responds to TH signaling [34]. Moreover, thyroid glands of stage 48 tadpoles remain undeveloped and thereby tadpoles theoretically have little capability to respond to chemicals that directly or indirectly disrupt TH synthesis, especially within 24 h [25,26]. Therefore, we assure that the assay is relatively specific for screening TH signaling disruptors. Certainly, the results of the screening assay need further confirmation by other assays such as TIXMA. Importantly, the screening assay is more rapid in terms of the 24-h exposure duration relative to the 72-h exposure duration in XETA. In addition, the screening assay, like the XETA, is simpler and easier to implement relative to the TIXMA due to chemical exposure in 6-well plates. Moreover, the exposure duration of 24 h in our assay ensures more rapid detection for TH signaling disruption compared with the 72-h exposure duration in XETA. Additionally, Fini et al. (2007) [19] reported that 1000 nM TBBPA inhibited T3-induced GFP signaling in transgenic TH/bZIP-GFP tadpoles using the XETA assay, but 500 nM failed. Here, we found that both 1000 nM and 100 nM TBBPA significantly antagonized T3-induced *thibz* expression. Therefore, the screening assay we developed is more sensitive or comparable with the XETA assay for detecting antagonistic actions of chemicals on TH signaling.

Using this screening assay, we examined several chemicals for TH signaling disrupting activity. In the Tox21 project, betamipron was for the first time reported as a TR agonist in the in vitro reporter gene assay [28], with the lack of in vivo data. Here, we found that betamipron significantly upregulated *thibz* expression even at 10 nM in our screening assay, providing the first in vivo evidence that betamipron is a TH signaling agonist. In contrast to betamipron, the screening assay revealed that BDE-47 inhibited T3-induced *thibz* expression as BDE-47 exposure alone had no effect, indicating TH signaling antagonism. Previous studies reported that BDE-47 inhibited TH-dependent development in *X. laevis* [35,36], and decreased the expression of the TH-response genes *thrb* and *klf9* in *X. laevis* [35]. Together with these data, our findings support that BDE-47 could have the potential to antagonize TH signaling, warranting further investigations.

In the screening assay, TCC, TCS, BP, and BP-3 exerted similar effects on *thibz* expression in either the absence or presence of T3, i.e., they stimulated *thibz* gene expression in the absence of T3 but antagonized T3-induced expression in the presence of T3. The effects of these chemicals are similar to those of BPA and TBBPA, implying that they, like BPA and TBBPA, exerted TH signaling antagonistic action in the presence of T3, but could have agonistic action in the absence of T3. Previously, several studies reported that TCS altered TH-response gene expression and TH-dependent growth in amphibians, despite seemly inconsistent effects [37,38,39]. Similarly, BP-3 was reported to inhibit T3-induced tail resorption in *Rana rugose* and suppressed the T3-induced EGFP activity in transgenic *X. laevis* tadpoles [32], but behaved as TR agonists in HepG2 cells on the activation of TR-mediated transcription [40]. Given these data combined with our results from the screening assay, it is concluded that TCC, TCS, BP, and BP-3 could be TH signaling disruptors, which warrants further studies, including the investigation of the mechanisms for TH signaling disruption.

## 4. Materials and Methods

### 4.1. Chemicals

3,3′,5-triiodo-L-thyronine (T3, CAS No. 6893-02-3) was obtained from Geel Belgium (New Jersey, USA) and a stock solution of T3 was prepared by dissolving into ultrapure water. Dimethyl sulfoxide (DMSO, CAS No. 67-68-5) and 3-aminobenzoic acid ethyl ester (MS-222, CAS No. 886-86-2) were purchased from Sigma-Aldrich (St. Louis, MO, USA). Bisphenol A (BPA, CAS No. 80-05-7, Acros Organics, Geel, Belgium), tetrabromobisphenol A (TBBPA, CAS No. 79-94-7, Acros Organics, Geel, Belgium), betamipron (CAS No. 3440-28-6, Aladdin, Shanghai, China), 2,2′,4,4′-tetrabromodiphenyl ether (BDE-47, CAS No. 5436-43-1, Bidepharm, Shanghai, China), triclocarban (TCC, CAS No. 101-20-2, Tokyo Chemical Industry, Tokyo, Japan), triclosan (TCS, CAS No. 3380-34-5, Tokyo Chemical Industry, Tokyo, Japan), benzophenone (BP, CAS No. 119-61-9, Macklin, Shanghai, China) and benzophenone-3 (BP-3, CAS No. 131-57-7, Tokyo Chemical Industry, Tokyo, Japan) were dissolved into DMSO to prepare 10 mM stock solutions. The stock solutions for chemicals were stored at −20 °C for future exposure experiments. An RNA Extraction kit (AU1201) was obtained from BioTeke Corporation (Wuxi, China). PCR primers were synthesized by BGI Tech Solutions (Beijing, China). DNase/RNase-free water (RT121), Quantscript RT Kit (KR116), and a Real Master Mix (SYBR Green) Kit (FP205) were obtained from TIANGEN Biotech (Beijing, China). Human chorionic gonadotropin (HCG) was purchased from the Ningbo Second Hormone Factory (Ningbo, China) and dissolved into 0.6% sodium chloride.

### 4.2. Animals and Housing Conditions

Housing and breeding conditions for *X. laevis* were described in our previous study with some adjustments [41]. In brief, sexually mature wild-type adult *X. laevis* were raised at 20 °C with a light/dark cycle of 12 h:12 h in charcoal-filtered tap water. Breeding was induced by injection HCG into a pair of adult frogs (800 IU for the female and 400 IU for the male). After spawning, fertilized eggs were incubated in glass aquariums containing charcoal-filtered tap water with a 12 h light/12 h dark cycle at 22 ± 1 °C. Developmental stages of *Xenopus* tadpoles were identified according to the Nieuwkoop and Faber table [26]. This study was approved by Animal Ethics and Welfare Committee of Research Center for Eco-Environmental Sciences, Chinese Academy of Sciences (AEWC-RCEES-2021040).

### 4.3. Chemical Exposure and Sampling

To reduce the test size, we employed stage 48 tadpoles in 6-well plates in place of stage 52 tadpoles in glass tanks in the TIXMA. Our previous study has shown that stage 48 tadpoles are highly sensitive to T3 [42]. To determine an appropriate concentration of T3 that can induce TH-response gene expression, a concentration-response experiment (0, 0.5, 1, 2, and 5 nM) was performed. Three replicate wells were set for each treatment, with two tadpoles per well containing 10 mL of the test solution. After 24 h, tadpoles were anesthetized with 100 mg/L MS-222 and then rinsed with water. Tadpoles (tails removed) in each well were pooled for RNA extraction and subsequent analysis for TH-response gene expression. Following the TIXMA, six TH-response genes were chosen, including *klf9*,* thibz*,* thrb*,* st3*,* dio3*, and *mmp13* [21]. Then, stage 48 tadpoles were exposed to BPA and TBBPA (1, 10, 100, and 1000 nM) in the presence or absence of T3 in order to verify the response of this assay to TH signaling antagonists. The most sensitive gene to T3, BPA, and TBBPA was chosen as the endpoint for screening TH signaling disruptors.

Finally, stage 48 tadpoles were exposed to betamipron, BDE-47, TCC, TCS, BP, and BP-3 (1, 10, 100, and 1000 nM) in the absence or presence of T3 to screen their TH signaling disrupting activity. Exposures and sampling were conducted as described above. Each experiment was independently repeated three times using offspring from different pairs of *X. laevis*.

### 4.4. RNA Extraction and Quantitative Real-Time PCR

For gene expression analysis, the total RNA of tadpoles was extracted by an Automatic Nucleic Acid Extraction Apparatus (BioTeke, Wuxi, China) following the manufacturer’s instructions. RNA concentration was measured using a NanoDrop 2000 (Thermo Scientific, Waltham, MA, USA)*,* and RNA quality was examined by A260/A280 ratios and agar-gel electrophoresis. The first-strand cDNA was synthesized from 1000 ng total RNA using the Fast RT Kit according to the manufacturer’s instructions (TIANGEN Biotech, Beijing, China). Following this, the first-strand cDNA was stored at −20 °C for gene expression analysis. Expression of TH-response genes was analyzed using SYBR Green I with the Real-time Polymerase Chain Reaction system (Light Cycler 480, Roche, Basel, Switzerland), with *Ribosomal protein L8* (*rpl8*) as a reference gene [43,44,45] and the expression of *rpl8* was not affected by treatments (Supplementary Materials, Figures S1 and S2). In 10 µL of PCR reaction system, 1 µL of cDNA template, 0.3 µL of forward primer, 0.3 µL of reverse primer, 3.4 µL of DNase/RNase-free water, and 5 µL of 2 × SuperReal PreMix Plus were mixed. All primers and conditions for PCR are listed in Table 1. The relative fold changes of targeted gene expression data using real-time PCR were calculated using the 2^−ΔΔCt^ methods [46].

### 4.5. Statistical Analysis

The statistical analysis of the experimental data was performed using SPSS software version 16.0 (IBM, Armonk, NY, USA). The data were checked for normal distribution (Kolmogorov–Smirnov test) and homogeneity of variance (Levene test). Data for relative expression of genes are presented as mean ± standard error of the mean (SEM). Statistical differences in relative expression of genes were analyzed by two-way analysis of variance (ANOVA) followed by Tukey’s HSD test. Statistical significance was defined as *p* value < 0.05. 

## 5. Conclusions

We developed a multiwell-based assay for rapidly screening TH signaling disruptors using thibz expression as a sensitive endpoint in X. laevis which effectively detected the agonistic effect of T3 and the antagonistic effect of BPA and TBBPA on TH signaling within 24 h. Using this screening assay, we identified betamipron as a TH signaling agonist and BDE-47 as a TH signaling antagonist, while TCC, TCS, BP, and BP-3 appeared to exert complex TH signaling disrupting actions in a TH-dependent manner. Overall, all results indicate that this assay is suitable for screening the TH signaling disrupting activity of chemicals.

## Figures and Tables

**Table 1:** Primer sequences of all tested *Xenopus laevis* genes and conditions for quantitative polymerase chain reaction (qPCR).

Gene	Primer Sequences (5′–3′)	Annealing Temperature (°C)	GeneBank ID
*rpl8*	F: CCGTGGTGTGGCTATGAATC	58	NM_001086996.1
	R: TACGACGAGCAGCAATAAGAC		
*klf9*	F: GTGGCCACTTGATTTCCCCT	64	NM_001085597.1
	R: AAAGACACAAAACAGCGGCG		
*thibz*	F: CCACCTCCACAGAATCAGCAG	62	NM_001085805.1
	R: AGAAGTGTTCCGACAGCCAAG		
*thrb*	F: GAGATGGCAGTGACAAGG	58	NM_001087781.1
	R: CAAGGCGACTTCGGTATC		
*st3*	F: CCTCTGTCATACACTTACCTT	62	NM_001086342.1
	R: TGAACCGTGAGCATTGAG		
*dio3*	F: GATGCTGTGGCTGCTGGAT	62	NM_001087863.1
	R: ATTCGGTTGGAGTCGGACAC		
*mmp13*	F: CCTTGTCAGTGCTTGTCCTATC	62	NM_001100931.1
	R: TCCTGGTGTCAGTTCAGAGTC		

F: forward; R: reverse; *rpl8: ribosomal protein L8; klf9: Kruppel-like factor 9; thibz: thyroid hormone induced bZip protein; thrb: thyroid hormone receptor beta; st3: stromelysin-3; dio3: type 3 deiodinase; mmp13: matrix metallopeptidase 13*.

## Data Availability

The authors declare that all data generated or analyzed during this study are included in the published article.

## Supplementary Materials

The following are available online, Figure S1: Relative expression of ribosomal protein L8 (rpl8) in stage 48 *Xenopus* tadpoles following 24-h exposure to T3. Figure S2: Relative expression of rpl8 in stage 48 *Xenopus* tadpoles following 24-h exposure to betamipron, 2,2′,4,4′-tetrabromodiphenyl ether (BDE-47), triclocarban (TCC), triclosan (TCS), benzophenone (BP), and benzophenone-3 (BP-3) in the absence or presence of 1 nM T3.

## References

[1] Sachs L.M., Campinho M.A. (2019). Editorial: The Role of Thyroid Hormones in Vertebrate Development. Front. Endocrinol..

[2] Shi Y.B. (2009). Dual functions of thyroid hormone receptors in vertebrate development: The roles of histone-modifying cofactor complexes. Thyroid.

[3] Yen P.M., Ando S., Feng X., Liu Y., Maruvada P., Xia X.M. (2006). Thyroid hormone action at the cellular, genomic and target gene levels. Mol. Cell. Endocrinol..

[4] DeVito M., Biegel L., Brouwer A., Brown S., Brucker-Davis F., Cheek A.O., Christensen R., Colborn T., Cooke P., Crissman J. (1999). Screening methods for thyroid hormone disruptors. Environ. Health Perspect..

[5] Zhang J., Li Y.Z., Gupta A.A., Nam K., Andersson P.L. (2016). Identification and Molecular Interaction Studies of Thyroid Hormone Receptor Disruptors among Household Dust Contaminants. Chem. Res. Toxicol..

[6] Leemans M., Couderq S., Demeneix B., Fini J.B. (2019). Pesticides with Potential Thyroid Hormone-Disrupting Effects: A Review of Recent Data. Front. Endocrinol..

[7] Gilbert M.E., O’Shaughnessy K.L., Axelstad M. (2020). Regulation of Thyroid-disrupting Chemicals to Protect the Developing Brain. Endocrinology.

[8] Moriyama K., Tagami T., Akamizu T., Usui T., Saijo M., Kanamoto N., Hataya Y., Shimatsu A., Kuzuya H., Nakao K. (2002). Thyroid hormone action is disrupted by bisphenol A as an antagonist. J. Clin. Endocr. Metab..

[9] Sun H., Shen O.X., Wang X.R., Zhou L., Zhen S.Q., Chen X.D. (2009). Anti-thyroid hormone activity of bisphenol A, tetrabromobisphenol A and tetrachlorobisphenol A in an improved reporter gene assay. Toxicol. In Vitro.

[10] Niu Y., Zhu M., Dong M., Li J., Li Y., Xiong Y., Liu P., Qin Z. (2021). Bisphenols disrupt thyroid hormone (TH) signaling in the brain and affect TH-dependent brain development in *Xenopus laevis*. Aquat. Toxicol..

[11] Zhang Y.F., Xu W., Lou Q.Q., Li Y.Y., Zhao Y.X., Wei W.J., Qin Z.F., Wang H.L., Li J.Z. (2014). Tetrabromobisphenol A disrupts vertebrate development via thyroid hormone signaling pathway in a developmental stage-dependent manner. Environ. Sci. Technol..

[12] Freitas J., Cano P., Craig-Veit C., Goodson M.L., Furlow J.D., Murk A.J. (2011). Detection of thyroid hormone receptor disruptors by a novel stable in vitro reporter gene assay. Toxicol. In Vitro.

[13] Hofmann P.J., Schomburg L., Kohrle J. (2009). Interference of Endocrine Disrupters with Thyroid Hormone Receptor-Dependent Transactivation. Toxicol. Sci..

[14] Zoeller R.T., Tyl R.W., Tan S.W. (2007). Current and potential rodent screens and tests for thyroid toxicants. Crit. Rev. Toxicol..

[15] Grimaldi M., Boulahtouf A., Delfosse V., Thouennon E., Bourguet W., Balaguer P. (2015). Reporter cell lines for the characterization of the interactions between human nuclear receptors and endocrine disruptors. Front. Endocrinol..

[16] Thambirajah A.A., Koide E.M., Imbery J.J., Helbing C.C. (2019). Contaminant and Environmental Influences on Thyroid Hormone Action in Amphibian Metamorphosis. Front. Endocrinol..

[17] Sachs L.M., Buchholz D.R. (2017). Frogs model man: In vivo thyroid hormone signaling during development. Genesis.

[18] OECD/OCDE (2009). Test No. 231: Amphibian Metamorphosis Assay. OECD Guidelines for the Testing of Chemicals, Section 2.

[19] Fini J.B., Le Mevel S., Turque N., Palmier K., Zalko D., Cravedi J.P., Demeneix B.A. (2007). An in vivo multiwell-based fluorescent screen for monitoring vertebrate thyroid hormone disruption. Environ. Sci. Technol..

[20] OECD/OCDE (2019). Test No. 248: Xenopus Eleutheroembryonic Thyroid Assay (XETA). OECD Guidelines for the Testing of Chemicals, Section 2.

[21] Yao X., Chen X., Zhang Y., Li Y., Wang Y., Zheng Z., Qin Z., Zhang Q. (2017). Optimization of the T3-induced *Xenopus* metamorphosis assay for detecting thyroid hormone signaling disruption of chemicals. J. Environ. Sci. (China).

[22] Wang Y., Li Y., Qin Z., Wei W. (2017). Re-evaluation of thyroid hormone signaling antagonism of tetrabromobisphenol A for validating the T3-induced *Xenopus* metamorphosis assay. J. Environ. Sci. (China).

[23] Yaoita Y., Brown D.D. (1990). A correlation of thyroid hormone receptor gene expression with amphibian metamorphosis. Genes Dev..

[24] Morvan-Dubois G., Demeneix B.A., Sachs L.M. (2008). *Xenopus laevis* as a model for studying thyroid hormone signalling: From development to metamorphosis. Mol. Cell. Endocrinol..

[25] Saxen L., Saxen E., Toivonen S., Salimaki K. (1957). Quantitative investigation on the anterior pituitary-thyroid mechanism during frog metamorphosis. Endocrinology.

[26] Faber J., Nieuwkoop P.D. (1994). Normal Table of Xenopus Laevis (Daudin). A Systematical and Chronological Survey of the Development from the Fertilized Egg till the End of Metamorphosis.

[27] Hirouchi Y., Naganuma H., Kawahara Y., Okada R., Kamiya A., Inui K., Hori R. (1994). Preventive Effect of Betamipron on Nephrotoxicity and Uptake of Carbapenems in Rabbit Renal-Cortex. Jpn. J. Pharmacol..

[28] Paul-Friedman K., Martin M., Crofton K.M., Hsu C.W., Sakamuru S., Zhao J., Xia M., Huang R., Stavreva D.A., Soni V. (2019). Limited Chemical Structural Diversity Found to Modulate Thyroid Hormone Receptor in the Tox21 Chemical Library. Environ. Health Perspect..

[29] Wu Y., Beland F.A., Fang J.-L. (2016). Effect of triclosan, triclocarban, 2,2′,4,4′-tetrabromodiphenyl ether, and bisphenol A on the iodide uptake, thyroid peroxidase activity, and expression of genes involved in thyroid hormone synthesis. Toxicol. In Vitro.

[30] Mihaich E., Capdevielle M., Urbach-Ross D., Slezak B. (2017). Hypothesis-driven weight-of-evidence analysis of endocrine disruption potential: A case study with triclosan. Crit. Rev. Toxicol..

[31] Lee J., Kim S., Park Y.J., Moon H.-B., Choi K. (2018). Thyroid Hormone-Disrupting Potentials of Major Benzophenones in Two Cell Lines (GH3 and FRTL-5) and Embryo-Larval Zebrafish. Environ. Sci. Technol..

[32] Kashiwagi K., Hanada H., Yamamoto T., Goto Y., Furuno N., Kitamura S., Ohta S., Sugihara K., Taniguci K., Tooi O. (2008). 2-Hydroxy-4-methoxybenzophenone (HMB) and 2,4,4′-trihydroxybenzophenone (THB) Suppress Amphibian Metamorphosis. Progress in Safety Science and Technology Series.

[33] Jiang Y., Yuan L., Lin Q., Ma S., Yu Y. (2019). Polybrominated diphenyl ethers in the environment and human external and internal exposure in China: A review. Sci. Total Environ..

[34] Furlow J.D., Brown D.D. (1999). In vitro and in vivo analysis of the regulation of a transcription factor gene by thyroid hormone during *Xenopus laevis* metamorphosis. Mol. Endocrinol..

[35] Yost A.T., Thornton L.M., Venables B.J., Jeffries M.K.S. (2016). Dietary exposure to polybrominated diphenyl ether 47 (BDE-47) inhibits development and alters thyroid hormone-related gene expression in the brain of *Xenopus laevis* tadpoles. Environ. Toxicol. Phar..

[36] Balch G.C., Velez-Espino L.A., Sweet C., Alaee M., Metcalfe C.D. (2006). Inhibition of metamorphosis in tadpoles of *Xenopus laevis* exposed to polybrominated diphenyl ethers (PBDEs). Chemosphere.

[37] Fort D.J., Mathis M.B., Pawlowski S., Wolf J.C., Peter R., Champ S. (2017). Effect of triclosan on anuran development and growth in a larval amphibian growth and development assay. J. Appl. Toxicol..

[38] Marlatt V.L., Veldhoen N., Lo B.P., Bakker D., Rehaume V., Vallee K., Haberl M., Shang D., van Aggelen G.C., Skirrow R.C. (2013). Triclosan exposure alters postembryonic development in a Pacific tree frog (*Pseudacris regilla*) Amphibian Metamorphosis Assay (TREEMA). Aquat. Toxicol..

[39] Veldhoen N., Skirrow R.C., Osachoff H., Wigmore H., Clapson D.J., Gunderson M.P., Van Aggelen G., Helbing C.C. (2006). The bactericidal agent triclosan modulates thyroid hormone-associated gene expression and disrupts postembryonic anuran development. Aquat. Toxicol..

[40] Schmutzler C., Bacinski A., Gotthardt I., Huhne K., Ambrugger P., Klammer H., Schlecht C., Hoang-Vu C., Grueters A., Wuttke W. (2007). The ultraviolet filter benzophenone 2 interferes with the thyroid hormone axis in rats and is a potent in vitro inhibitor of human recombinant thyroid peroxidase. Endocrinology.

[41] Lou Q.Q., Zhang Y.F., Zhou Z., Shi Y.L., Ge Y.N., Ren D.K., Xu H.M., Zhao Y.X., Wei W.J., Qin Z.F. (2013). Effects of perfluorooctanesulfonate and perfluorobutanesulfonate on the growth and sexual development of *Xenopus laevis*. Ecotoxicology.

[42] Zhang Y. (2014). Methods for Evaluating Thyroid Disruption by Chemicals Using Amphibians and Their Application. Ph.D. Thesis.

[43] Shi Y.B., Liang V.C.-T. (1994). Cloning and characterization of the ribosomal protein L8 gene from *Xenopus laevis*. Biochim. Biophys. Acta.

[44] Zhu M., Chen X.Y., Li Y.Y., Yin N.Y., Faiola F., Qin Z.F., Wei W.J. (2018). Bisphenol F Disrupts Thyroid Hormone Signaling and Postembryonic Development in *Xenopus laevis*. Environ. Sci. Technol..

[45] Crespi E.J., Denver R.J. (2006). Leptin (ob gene) of the South African clawed frog *Xenopus laevis*. Proc. Natl. Acad. Sci. USA.

[46] Livak K.J., Schmittgen T.D. (2001). Analysis of relative gene expression data using real-time quantitative PCR and the 2^−ΔΔCt^ method. Methods.

