# Supplementary material for: A Multiwell-Based Assay for Screening Thyroid Hormone Signaling Disruptors Using thibz Expression as a Sensitive Endpoint in Xenopus laevis
Source: Molecules. 2022 Jan 25;27(3):798. doi: 10.3390/molecules27030798 (PMC8838645; doi:10.3390/molecules27030798)
Supplement: Supplementary file 1 [file molecules-27-00798-s001.zip › molecules-1530770-supplementary.pdf]

Supplementary Materials

A Multiwell-Based Assay for Screening Thyroid Hormone Signaling Disruptors Using thibz Expression as a Sensitive Endpoint in Xenopus laevis

Jinbo Li [1,2], Yuanyuan Li [1,2], Min Zhu [1,2], Shilin Song [1,2] and Zhanfen Qin [1,2,*]

[1] State Key Laboratory of Environmental Chemistry and Ecotoxicology, Research Center for Eco-Environmental Sciences, Chinese Academy of Sciences, Beijing, 100085, China; jbli2016_st@rcees.ac.cn (J.L.); yyli@rcees.ac.cn (Y.L.); zhumin2@jshb.gov.cn (M.Z.); songshilin21@mails.ucas.ac.cn (S.S.)

[2] University of Chinese Academy of Sciences, Beijing, 100049, China

* Correspondence: qinzhanfen@rcees.ac.cn; Tel.: +86-10-6291-9177; Fax: +86-10-6292-3563.

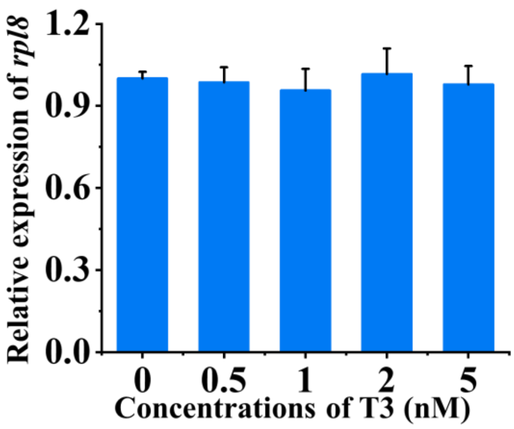

Figure S1. Relative expression of *rpl8* in stage 48 *Xenopus* tadpoles following 24-h exposure to T3. Data are shown as mean ± SEM (*n* = 3). No significant difference was found between T3 treatments and controls on the expression of *rpl8*.

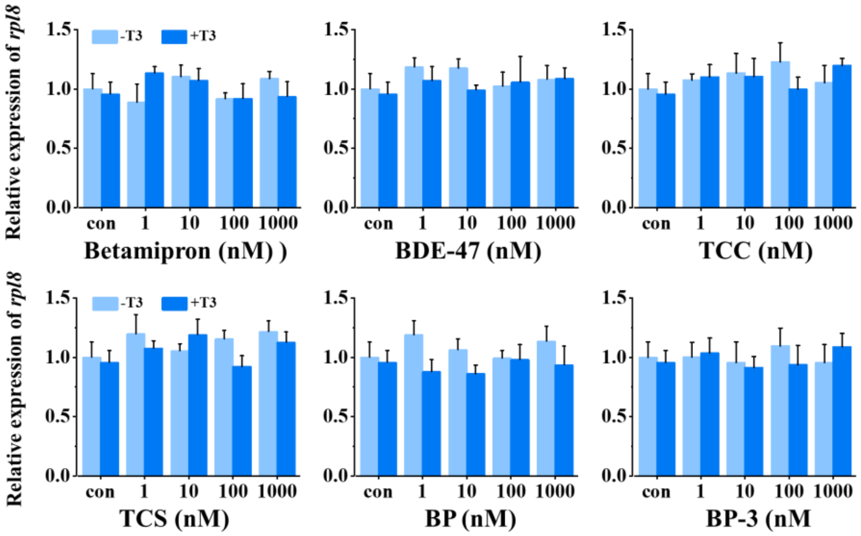

Figure S2. Relative expression of *rpl8* in stage 48 *Xenopus* tadpoles following 24-h exposure to betamipron, 2,2′,4,4′-tetrabromodiphenyl ether (BDE-47), triclocarban (TCC), triclosan (TCS), benzophenone (BP), and benzophenone-3 (BP-3) in the absence or presence of 1 nM T3. Data are shown as mean ± SEM (*n* = 3). Those chemicals treatment alone or treatment with 1 nM T3 didn't significantly affect the expression of *rpl8*.
